# Neutron and X-ray powder diffraction data to determine the structural properties of novel layered perovskite PrSrMn_2_O_5+δ_

**DOI:** 10.1016/j.dib.2020.105173

**Published:** 2020-01-25

**Authors:** Shammya Afroze, Nico Torino, Paul F. Henry, Md Sumon Reza, Quentin Cheok, Abul K. Azad

**Affiliations:** a Faculty of Integrated Technologies, Universiti Brunei Darussalam, Jalan Tungku Link, Gadong, BE 1410, Brunei Darussalam; b Department of Chemistry and Chemical Engineering, Chalmers University of Technology, SE-412 96 Gothenburg, Sweden; c ISIS Pulsed Neutron & Muon Facility, Rutherford Appleton Laboratory, Harwell Campus, OX11 0QX, United Kingdom

**Keywords:** Perovskite oxide, PrSrMn_2_O_5+δ_, Neutron powder diffraction, X-ray diffraction

## Abstract

The data presented in this article are related to the formation of a novel layered perovskite oxide material, PrSrMn_2_O_5+δ_, through a solid-state synthesis route. Here, we present the high-resolution neutron powder diffraction and the X-ray powder diffraction data at room temperature. The new perovskite material crystallizes in the orthorhombic symmetry. Interpretation of this data can be found in a research article titled “Insight of novel layered perovskite PrSrMn2O5+δ: A neutron powder diffraction study” (Shammya et *al*., 2019) [1].

**Table:** Specifications Table

Subject area	Material science
More specific subject area	Layered perovskite-type oxide - ceramic Material
Type of data	Figure, raw data and analyzed data, table
How data was acquired	Neutron powder diffraction data were obtained on a Polaris instrument, the X-ray diffraction (XRD) data were collected on a Bruker AXS D8 Advance diffractometer.
Data format	Raw (neutron data:.gsas), txt and dat
Experimental factors	Powder sample
Experimental features	Neutron powder diffraction experiment was started when pressure measured approximately 1 mbar. Neutron data on 90° bank (up to 4.1 Å) was collected over one hour at room temperature. For the X-ray powder diffraction, the sample was measured over a 2θ interval from 10° to 79.995° with a step size of 0.02
Data source location	High-resolution neutron powder diffraction data were collected on the time-of-flight instrument Polaris at the ISIS Pulsed Neutron & Muon Source Institution: Rutherford Appleton Laboratory City/Town/Region: Harwell Campus, OX11 0QX Country: United Kingdom and the XRD data were collected at Department of Chemistry and Chemical Engineering Laboratory Institution: Chalmers University of Technology City/Town/Region: SE-412 96 Gothenburg Country: Sweden
Data accessibility	Data is with the article
Related research article	Shammya Afroze, Nico Torino, Paul Henry, Md Sumon Reza, Quentin Cheok, Abul K. Azad, Insight of novel layered perovskite PrSrMn2O5+δ: A neutron powder diffraction study, Materials Letters, DOI: 10.1016/j.matlet.2019.127126 [1]

**Table:** 

**Value of the data** • The data provides detailed information on how to investigate crystal symmetry, space group, lattice parameter, atomic positions of a layered-type perovskite oxide material. • Data to be used on understanding to observe its structural properties using a range of techniques. • The method and structural model analysis are worthy of being applied to other types of perovskite-type oxide materials.

## 1 Data

**Fig. 1:**
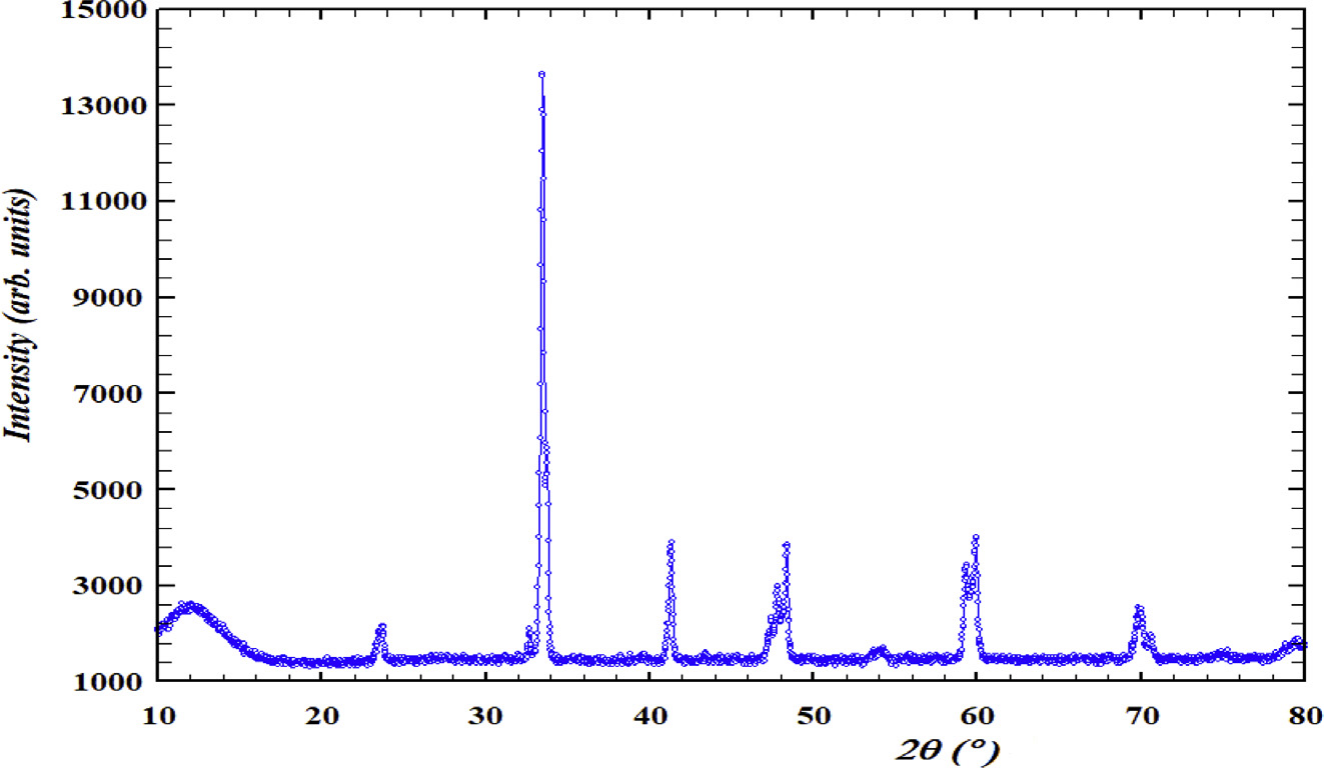
Raw XRD pattern of PrSrMn_2_O_5+δ_ composition sintered at 1400 °C for 12 h in Ar.

**Fig. 2:**
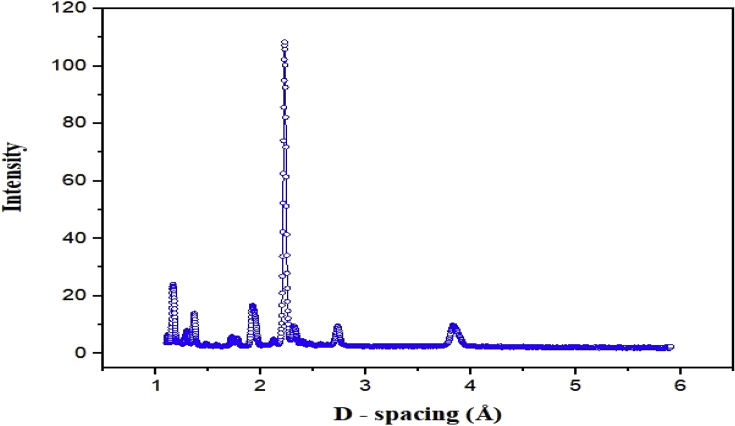
Raw neutron powder diffraction data collected on 90° bank at room temperature.

The new layered perovskite material, PrSrMn_2_O_5+δ_, was synthesized by solid-state reaction to investigate the structural behavior. X-ray and neutron powder diffraction data were presented at room temperature in Fig. 1, Fig. 2. The XRD pattern of the sample was shown the same crystalline nature of the ceramic material. The XRD pattern was obtained at room temperature for the above sample. To understand the structure of the sample behavior, neutron powder diffraction was also carried out on PrSrMn_2_O_5+δ_ sample at room temperature. A small impurity phase was detected and the percentage of impurity was ∼2% for MnO_2_. The neutron diffraction pattern is perfectly fitted with the orthorhombic layered perovskite structure in the *Pmmm* space-group yielding, a = 3.8907 (1) Å, b = 3.8227 (1) Å, and c = 7.6846 (2) Å, with dimensions a_p_ × a_p_ × 2a_p_. The dimensions were chosen on the basis of X-ray and neutron powder diffraction studies.

The impurity phase for MnO_2_ also obtained the same crystalline symmetry (orthorhombic symmetry with space group, Pnma). The XRD and neutron diffraction patterns are perfectly matched with cell parameter, a = 9.2451 (1) Å, b = 3.1108 (1) Å and c = 4.3475 (2) Å. What are also presented in the article are the detailed neutron powder diffraction data and atomic coordinates (Table 1).

**Table 1:** Structural parameters for as-prepared PrSrMn_2_O_5+δ_ at RT with orthorhombic structure.

Parameters	PrSrMn_2_O_5+δ_ at RT
Structure model	PrSrMn_2_O_5+δ_
Crystal system	Orthorhombic
Space group	*Pmmm*
Volume (Å^3^)	480.9290 (0)
Density (gm/cm^3^)	6.9870 (1)
**Cell parameters**	
a (Å)	3.8906 (1), α = 90°
b (Å)	3.8227 (1), β = 90°
c (Å)	7.6846 (2), γ = 90°
**Atomic positions**	
Pr (x, y, z)	(0.5000, 0.5000, 0.0000)
Sr (x, y, z)	(0.5000, 0.5000, 0.5000)
Mn (x, y, z)	(0.0000, 0.0000, 0.7547)
O1 (x, y, z)	(0.0000, 0.5000, 0.2522)
O2 (x, y, z)	(0.5000, 0.0000, 0.2517)
O3 (x, y, z)	(0.0000, 0.0000, 0.5000)
Structure model	MnO_2_
Crystal system	Orthorhombic
Space group	*Pnma*
Volume (Å^3^)	131.7360 (0)
Density (gm/cm^3^)	1.7500 (1)
**Cell parameters**	
a (Å)	9.2451 (1), α = 90°
b (Å)	3.1108 (1), β = 90°
c (Å)	4.3475 (2), γ = 90°
**Atomic positions**	
Mn (x, y, z)	(0.1545, 0.7500, 0.9957)
O1 (x, y, z)	(0.0128, 0.2500, 0.6998)
O2 (x, y, z)	(0.2613, 0.2500, 0.3399)

## 2 Experimental design, materials, and methods

### 2.1 Materials and methods

PrSrMn_2_O_5+δ_ was prepared by solid-state reaction, using carbonate and oxides: Pr_6_O_11_ (≥99.99%, Aldrich), SrCO_3_ (≥99.9%, Aldrich) and MnO (≥99.5%, Aldrich). The obtained powders were annealed at 1000 °C for 10 hours. Stoichiometric mixtures were prepared by manually grinding the reactants in an agate mortar-pestle, with ethanol as a suspending agent. The finely mixed powders were pressed into pellets and fired at 1200 °C in α-alumina crucibles for 12 hrs, then intensively grounded and pelletized again. The pellet was finally re-sintered for another 12 hrs at 1400 °C, with intermediate grinding and pelletizing. The samples were exposed to a stepwise temperature programme, using the method described in a previous study [2,3].

### 2.2 Neutron powder diffraction

Neutron powder diffraction data were collected on the time-of-flight instrument Polaris at the ISIS neutron and muon source, UK [4]. The samples were loaded into open, cylindrical 8mm external diameter vanadium can. Time-of-flight powder diffraction data were obtained using the raw format and analyzed on GSAS-II [5] software. The experiments were carried out under vacuum, while pressure was controlled by an inlet and outlet valve.

### 2.3 X-ray diffraction

X-ray powder diffraction (XPD) analysis was performed on a Bruker AXS D8 Advance diffractometer (Cu K radiation – λ = 1.54056 Å). The experiment was conducted with a 0.02° step, between 10° and 79.995°. The instrument equipped with a copper target, a Ge (111) primary monochromator, and a solid-state LynxEye detector. The powder diffraction patterns for PrSrMn_2_O_5+δ_ was generated using the software Fullprof.

## CRediT author statement

**Shammya Afroze:** Sample preparation, Characterization, Writing.: **Nico Torino**: Data curation.: **Paul Henry:** Data curation, Data analysis.: **Sumon Reza:** Writing, Conceptualization, Methodology.: **Quentin Cheok:** Data curation, Writing- Original draft preparation.: **Abul Azad:** Conceptualization, Investigation, Supervision.
